# Users' Perspective on the AI-Based Smartphone PROTEIN App for Personalized Nutrition and Healthy Living: A Modified Technology Acceptance Model (mTAM) Approach

**DOI:** 10.3389/fnut.2022.898031

**Published:** 2022-07-01

**Authors:** Sofia Balula Dias, Yannis Oikonomidis, José Alves Diniz, Fátima Baptista, Filomena Carnide, Alex Bensenousi, José María Botana, Dorothea Tsatsou, Kiriakos Stefanidis, Lazaros Gymnopoulos, Kosmas Dimitropoulos, Petros Daras, Anagnostis Argiriou, Konstantinos Rouskas, Saskia Wilson-Barnes, Kathryn Hart, Neil Merry, Duncan Russell, Jelizaveta Konstantinova, Elena Lalama, Andreas Pfeiffer, Anna Kokkinopoulou, Maria Hassapidou, Ioannis Pagkalos, Elena Patra, Roselien Buys, Véronique Cornelissen, Ana Batista, Stefano Cobello, Elena Milli, Chiara Vagnozzi, Sheree Bryant, Simon Maas, Pedro Bacelar, Saverio Gravina, Jovana Vlaskalin, Boris Brkic, Gonçalo Telo, Eugenio Mantovani, Olga Gkotsopoulou, Dimitrios Iakovakis, Stelios Hadjidimitriou, Vasileios Charisis, Leontios J. Hadjileontiadis

**Affiliations:** ^1^CIPER, Faculdade de Motricidade Humana, Universidade de Lisboa, Estrada da Costa, Lisbon, Portugal; ^2^Intrasoft International SA, Thessaloniki, Greece; ^3^Grupo CMC, Madrid, Spain; ^4^Centre for Research and Technology Hellas, Thessaloniki, Greece; ^5^Institute of Applied Biosciences, Centre for Research and Technology Hellas, Thessaloniki, Greece; ^6^School of Biosciences and Medicine, Faculty of Health and Medical Sciences, University of Surrey, Guildford, United Kingdom; ^7^OCADO Technology, London, United Kingdom; ^8^Department of Endocrinology, Diabetes and Nutrition and German Institute of Human Nutrition, Charité, Universitätsmedizin Berlin, Berlin, Germany; ^9^Department of Nutritional Sciences and Dietetics, International Hellenic University, Thessaloniki, Greece; ^10^Department of Rehabilitation Sciences and Department of Cardiovascular Sciences, Katholieke Universiteit Leuven, Leuven, Belgium; ^11^Sport Lisboa Benfica Futebol, Lisbon, Portugal; ^12^Polo Europeo della Conoscenza, Verona, Italy; ^13^Fluviale SRL, Rome, Italy; ^14^European Association for the Study of Obesity (EASO), Middlesex, United Kingdom; ^15^AgriFood Capital BV, Hertogenbosch, Netherlands; ^16^Healthium/Nutrium Software, Porto, Portugal; ^17^Datawizard SRL, Rome, Italy; ^18^BioSense Institute, Research and Development Institute for Information Technology in Biosystems, Novi Sad, Serbia; ^19^PLUX, Wireless Biosignals, Lisbon, Portugal; ^20^Research Group on Law, Science, Technology and Society, Faculty of Law & Criminology, Vrije Universiteit Brussel, Ixelles, Belgium; ^21^Department of Electrical and Computer Engineering, Aristotle University of Thessaloniki, Thessaloniki, Greece; ^22^Department of Biomedical Engineering, Healthcare Engineering Innovation Center (HEIC), Khalifa University of Science and Technology, Abu Dhabi, United Arab Emirates

**Keywords:** smartphone app-based nutrition support, AI-based personalized nutrition, healthy living, PROTEIN app, mobile application, modified Technology Acceptance Model (mTAM), behavior change

## Abstract

The ubiquitous nature of smartphone ownership, its broad application and usage, along with its interactive delivery of timely feedback are appealing for health-related behavior change interventions *via* mobile apps. However, users' perspectives about such apps are vital in better bridging the gap between their design intention and effective practical usage. In this vein, a modified technology acceptance model (mTAM) is proposed here, to explain the relationship between users' perspectives when using an AI-based smartphone app for personalized nutrition and healthy living, namely, PROTEIN, and the mTAM constructs toward behavior change in their nutrition and physical activity habits. In particular, online survey data from 85 users of the PROTEIN app within a period of 2 months were subjected to confirmatory factor analysis (CFA) and regression analysis (RA) to reveal the relationship of the mTAM constructs, i.e., perceived usefulness (PU), perceived ease of use (PEoU), perceived novelty (PN), perceived personalization (PP), usage attitude (UA), and usage intention (UI) with the users' behavior change (BC), as expressed *via* the acceptance/rejection of six related hypotheses (H1–H6), respectively. The resulted CFA-related parameters, i.e., factor loading (FL) with the related *p*-value, average variance extracted (AVE), and composite reliability (CR), along with the RA results, have shown that all hypotheses H1–H6 can be accepted (*p* < 0.001). In particular, it was found that, in all cases, FL > 0.5, CR > 0.7, AVE > 0.5, indicating that the items/constructs within the mTAM framework have good convergent validity. Moreover, the adjusted coefficient of determination (*R*^2^) was found within the range of 0.224–0.732, justifying the positive effect of PU, PEoU, PN, and PP on the UA, that in turn positively affects the UI, leading to the BC. Additionally, using a hierarchical RA, a significant change in the prediction of BC from UA when the UI is used as a mediating variable was identified. The explored mTAM framework provides the means for explaining the role of each construct in the functionality of the PROTEIN app as a supportive tool for the users to improve their healthy living by adopting behavior change in their dietary and physical activity habits. The findings herein offer insights and references for formulating new strategies and policies to improve the collaboration among app designers, developers, behavior scientists, nutritionists, physical activity/exercise physiology experts, and marketing experts for app design/development toward behavior change.

## Introduction

Unhealthy diets and physical inactivity are considered among the main modifiable risk factors for non-communicable diseases, the so-called Lifestyle Diseases. In fact, annually, 4.1 million deaths have been attributed to excess salt/sodium intake, and 1.6 million deaths attributed to physical inactivity (1). Many conventional approaches have been proposed and utilized to assess dietary intake and physical activity levels, mainly based on paper-based diaries and questionnaires (2, 3). However, inaccuracies and responder burden are common in observational dietary and physical activity assessments, alongside a significant degree of under-reporting that it is positively correlated with notable increased body mass index (BMI) (4). Fortunately, the advancement of technology has led to the development of innovative dietary and physical activity assessment methods, a key example is the use of nutrition/physical activity-related apps, which are readily available to access and use on smartphones or tablets (5, 6). Potential benefits that arise from these methods are the ease of use, convenience, and the wide availability of these apps *via* free downloading from app stores. Moreover, these mobile apps may also enable greater self-monitoring and disease management by individuals with chronic diseases such as obesity, cardiovascular disease, osteoporosis, and type 2 diabetes (7–10).

Mobile health apps, designed to support and reinforce health behaviors or to reduce risk behaviors, are among the most commonly downloaded apps, since they offer a novel and effective way for health professionals to engage users in the promotion and adoption of new behaviors (11, 12). Several mobile apps have been designed and developed specifically to increase physical activity levels (13, 14), encourage weight loss through personal coaching (15, 16), boost healthy food choices (17–20), reduce sedentary behavior (21, 22), assess and track dietary intake (23), assess psychological distress (e.g., anxiety, depression) (24), support smoke cessation (25), and reduce alcohol consumption (26). Moreover, mobile apps developed based on behavior change models and theories, including the integration of different behavior change strategies (e.g., cognitive behavioral therapy, goal setting, real time feedback), are considered the most effective in securing behavior change in their users (27, 28). Nevertheless, many apps designed for the purpose of changing behavior do not always involve input from health professionals and/or academics (29). From this perspective, it is clear that the adoption of the behavior change model alone is not sufficient and health professionals need also to consider how the mobile app is used to engage the user and, consequently, to facilitate behavior change. For instance, the Technology Acceptance Model (TAM), based on the Theory of Planned Behavior (TPB) (30) and originally proposed by Davis (31), has been widely used to evaluate user acceptance of general technologies. Overall, the TAM model investigates the drivers of technology acceptance, considering users' perceptions regarding innovations, and social/contextual aspects. Moreover, the validity of the TAM has been explored in different areas, such as wearable technologies (32) and telehealth (33), among others; however, limited explanatory power for particular system purposes has been identified (34).

Motivated by the aforementioned, this work aims to identify the main constructs and model their contributions to users' behavior change toward healthier living *via* personalized healthy diet and physical activity plans. To achieve this, the users' perspectives on the PROTEIN app (an artificial intelligence (AI)-based personalized nutrition mobile application for healthy living) are considered, and a modified Technology Acceptance Model (mTAM) is proposed. The main research problem that is examined here is whether specific interdependencies exist between the main constructs of a mTAM-based users' behavior model that could shed light upon the intention of the users to change their behavior and adopt a healthier living, undertaking personalized plans regarding their activity and nutrition, scaffolded by their interaction with the PROTEIN app.

## Theoretical Background

Successful use of information technology (IT) applications and what influences such success have been thoroughly explored, and great attention has been paying by the information systems (IS) community for the past decades (35–37) as proved by the considerable number of research articles published in major IS-related outlets (38). In this vein, several theories that contribute to better explain the adoption and use of IT application have emerged, examples of which include the TAM (31, 39) and the Unified Theory of Acceptance and Use of Technology (UTAUT) (40, 41). Both in TAM and UTAUT, the use of an IT application forms the dependent variable, i.e., the user's expected behavior when his/her working context (health status and lifestyle here) has been (or is about to be) affected by the introduction of an IT artifact (38). Subsequently, the independent variables refer to a wide range of factors, which have been known to influence the use behavior (42). Established theories from the field of social psychology, such as the TPB (30) and the Theory of Reasoned Action (TRA) (43), which explore the human behavior in general, were used to base both TAM and UTAUT. Acceptance, adoption, and use of IT applications are central behavioral constructs in TAM and UTAUT. Some related models have extended the perspective of the use of IT artifacts and focused on the success of IS, in terms of service, system, and information quality, with the DeLone and McLean's IS Success model (D&M-IS-SM) (44) being a milestone in such IS field. An epitomized description of the referenced models, i.e., TAM, UTAUT, and D&M-IS-SM, and their selection process follows.

### Technology Acceptance Model (TAM)

The TAM model, developed by Davis et al. (31, 39), is used to measure the acceptance, adoption, and use of IT and has become very popular with many validation studies (32, 33). It relies on five different constructs, namely, perceived usefulness (PU), perceived ease of use (PEoU), usage attitude (UA), usage intention (UI), and actual usage (AU). TAM does not focus on the success of the IT application, but on studying and predicting the user's intention to use it. TAM can be used for actual system use in both subjective (self-reporting questionnaire) and objective measurement (system log) (45). Different extensions have been proposed ever since its arrival, in an effort to strengthen TAM's explanatory power. Indicative examples include the work from: a) Liang and Yeh (46), who replaced the PU with the *Perceived Entertainment*, in an effort to understand if the users have different feelings when interacting with smartphone-based games across different settings and locations; b) Kim et al. (47), who added the construct of *Perceived Value*, to explore the system/service quality on users' beliefs of hospitality industry information management systems; c) Morosan (48), who added the construct of *Perceived Innovation*, when using TAM for exploring biometric systems utilization by hotels; d) Lee and Wan (49), who augmented TAM with the constructs of *Functional Confidence* and *Familiarity* for acceptance of airline e-ticketing services acceptance by travelers. These works show potential for TAM evolution; one of its referenced versions is TAM 3, which contemplates a comprehensive nomological network of the determinants of IT adoption and use by individuals (50). TAM has been tested and validated numerous times, and as Benbasat and Barki pointed out (51), TAM is considered as one of the most influential theories in IS.

### Unified Theory of Acceptance and Use of Technology (UTAUT)

A total of eight models already considered in, or resulting from, research on IT acceptance (40), are the combinatory basis for the formulation of the UTAUT, i.e., TRA (43); TAM (31, 39); motivational model (MM) (52, 53); TPB (30); a model combining TAM and the TPB (54); model of PC utilization (MPCU) (55); innovation diffusion theory (IDT) (56); and social cognitive theory (SCT) (57, 58). The behavioral intention is suggested by the UTAUT as the main factor that determines the use of IT application. A total of four key constructs have direct effect on the perceived likelihood of adopting the technology, i.e., *Performance Expectancy* (the degree to which a user believes that by using the IT application will gain attainment in his/her job performance), *Effort Expectancy* (the degree of ease associated with the use of the system), *Social Influence* (the degree to which a user is influenced by the opinions of important others, who believe that s/he should use the IT application), and *Facilitating Conditions* (the degree to which a user believes that an appropriate infrastructure, in terms of organization and technical perspectives, exists to support the use of the system) (40). Age, gender, experience, and voluntariness of use are considered as moderators of the effect of predictors (40, 59). The current version is UTAUT 2 (38, 41, 59), where the use of technology by individuals is underpinned by the effect of the three additional constructs, namely, *Hedonic Motive* (the fun or pleasure derived from using technology), *Cost/Perceived Value* (trade-off between perceived benefits and monetary cost of using the IT application), and *Habit* (the extent to which people tend to automatically perform behaviors) (41). The inclusion of hedonic motivation construct in the UTAUT 2 was found to be more important than performance expectancy (60), whereas the integration of price value provided a measure of the IS use cost within the consumer context (59).

### DeLone and McLean's IS Success Model (D&M-IS-SM)

D&M-IS-SM was first proposed in (44) and it is a process/causal model. It is based on Shannon and Weaver's model of communication (61, 62) and the extension by Mason (63). Actually, the idea behind was based on the assumption that the process in IS resembles the one in the communication system, considering IS as a process of information production, conveying it and transmitting it to the recipients/users. Motivated by the metrics of Shannon and Weaver's communication model, D&M-IS-SM was built upon three parts of instruments for measuring IS success, i.e., technical (system quality), semantic (information quality), and effectiveness success (use, user satisfaction, individual impact, and organizational impact) (44). D&M-IS-SM was criticized for the inclusion of the *Use* construct and thus was later revised (64, 65), where the *Service Quality* and *Use* constructs were replaced by the *Intention to Use/Use* and the *Individual Impact* and *Organizational Impact* were substituted by *Net Benefits*. The D&M-IS-SM serves a dual purpose: it provides a classification of success measures, and it shows how the measures influence each other. The D&M-IS-SM has numerously been tested and validated since its inception, with some studies showing the strong validity of the construct, whereas some others reporting partial validity only (66, 67).

### Technology Acceptance Framework Selection

As Madriana et al. (68) suggest, the selection of the suitable technology acceptance framework can be based on the availability of the variable(s) in the framework with evidence from previous studies that can be best predictors for the behavior intention, the validation of the framework (based on the popularity/citations), and the fecundity (“fertility” of the theory to generate new model and hypotheses). From a comparative analysis across the TAM, UTAUT, and D&M-IS-SM reported in (68), TAM exhibits the highest popularity and the most fecundity. With regard to the evidence from previous studies, TAM was preferred in evaluating users' adoption of mobile health apps for nutrition and active living, such as in (69–74). Furthermore, in the D&M-IS-SM, there is a very broad concept of *Net Benefits*; this poses some challenges in clearly and carefully defining the stakeholders and context in which such net benefits are to be measured (64, 65). Moreover, as D&M-IS-SM is focused on the quality of service, a consideration should be taken about the changes in the practice of service and in the conceptual view of service quality that the use of information technology introduced nowadays. The initial view of the service quality was on the services provided by the information system to its users. However, nowadays, the service is materialized *via* interactions between the user and the IT provider in a serving function; hence, the information and system quality are viewed as a resource owned by the IT provider, who can clearly influence the user's intentions to continue interacting with the IT provider (75).

Based on the aforementioned evidence, the TAM model has been selected here as a starting point to build a modified version (mTAM) and to explore the role of the PROTEIN app as the technology artifact that could help its users to change their behavior and adopt a healthier living, in terms of improved nutrition and activity.

## Materials and Methods

### The PROTEIN Smartphone App

Several digital mobile health technologies for self-management of nutrition (76) and physical activity (77) have been proposed in the digital market place. Nevertheless, a lack of expert nutrition and physical activity advice provided by current mobile apps is identified, igniting further concerns regarding personalized advice tailored to specific user preferences (including dietary and allergies, PA for heart health, etc.). For instance, a content analysis of more than 50 weight loss mobile apps showed that more than 60% of the apps lack expert recommendations (78). This fact leads to the demand for high-quality and evidence-based mobile app design/development process, considered as a co-creative process between designers, developers, researchers, experts/clinicians, and end-users (79).

**Figure 1:**
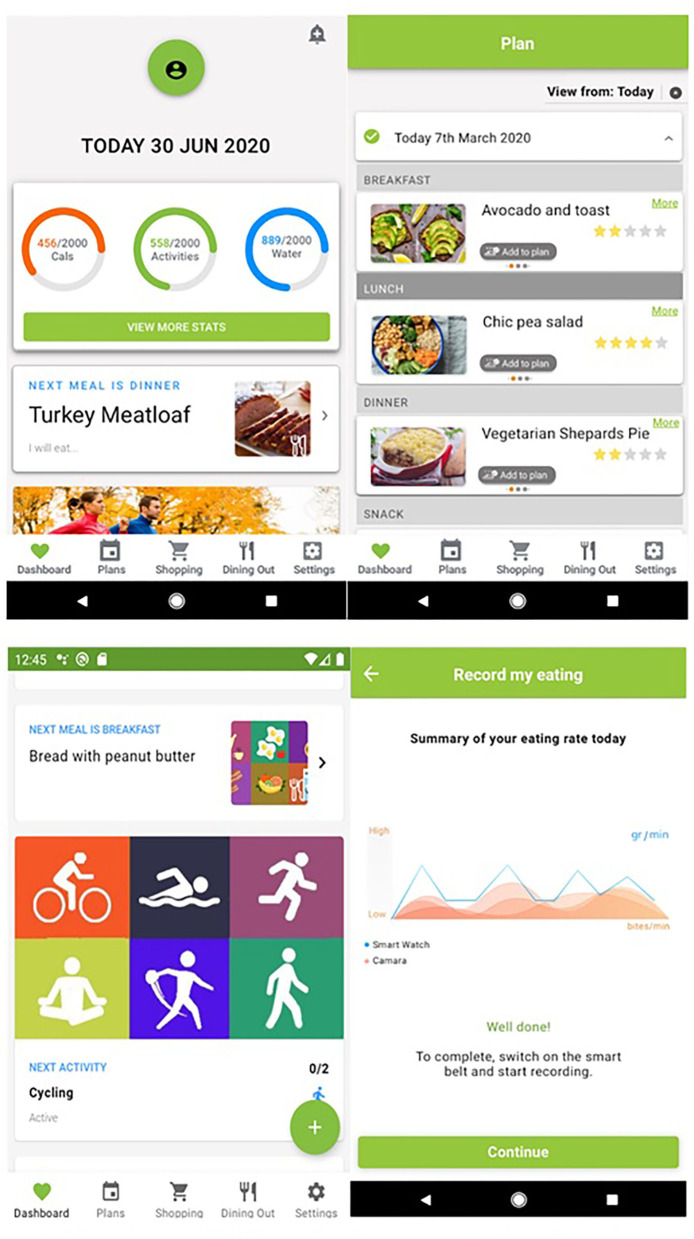
Screen shots of the PROTEIN mobile application [Source: Google Play Srore].

To address the aforementioned, a holistic approach is suggested with the introduction of the PROTEIN smartphone app. The latter is developed within the framework of an ongoing European H2020 research and innovation project (2019–2022), namely, “PROTEIN: PeRsOnalized nutriTion for hEalthy livINg” (https://protein-h2020.eu/). The latter involves a multidisciplinary team of 20 partners from a total of 11 European countries, including partners from industry, research, and technology organizations, aiming to promote a healthy lifestyle by combining the latest technologies, such as smartphones and sensors, to offer personalized nutrition and physical activity plans (80). Already available *via* Google Play Store^1^ and currently supporting eight different languages (i.e., English, Italian, Dutch, German, Portuguese, Greek, French, and Spanish), the PROTEIN app incorporates information provided in user profiles. This includes anthropometric, biochemical, physiological, and physical activity data and personal preferences and objectives, including eating and/or physical activity habits, and proposes personalized healthy-eating and physical activity advice (through personalized nutrition and physical activity plans) (Figure 1). An additional feature enables specialized advice to be provided to individuals living with or at risk of chronic diseases (e.g., obesity, cardiovascular disease, and diabetes). The PROTEIN app leverages automated decision-making tools, including profiling and AI, to propose advice on personalized meal plans and physical activities. In particular, by including a deep learning component to generate/update recommendations for the user's dietary intake (taking into consideration their dietary preferences, daily intake, lifestyle and physiological variables), the PROTEIN app provides recommendations for intakes within the expert-established targets (81). Furthermore, PROTEIN app supports additional functionalities, such as nearby restaurant recommendations, offering a range of price points and food types; customization of user's shopping cart using ingredients of eating plans proposed by the system, and incorporation of new meals into the system by recognizing images. This capability provides automated food volume estimation from just a single food image (82), facilitating the data input to the PROTEIN app and contributing toward the development of an accurate dietary assistant application. PROTEIN app acknowledges the effectiveness of health-related games to engage users in dealing with their health (83, 84) and informing them about deterioration of symptoms in diseases (85), and thus, it also incorporates different dietary games. In this way, PROTEIN app fosters adherence to the suggested plans, making the app fun to use, and providing the means for encouragement and/or (re)education on nutritional value of the various food groups toward the adoption of a healthy and balanced diet.

The PROTEIN app also provides a “Help center” portal for questions and/or suggestions to improve the functionalities of the PROTEIN app. However, it is important to underline that the PROTEIN app is not a medical device, meaning that information and advice that PROTEIN app provides does not constitute medical advice. Finally, complying with the General Data Protection Regulation (EU GDPR) (https://gdpr.eu), the PROTEIN app allows the users to download and have access to their data, withdraw from the application at any time and delete all previously collected data.

Since the PROTEIN app is co-designed with the users and implemented for their benefit, the analysis of their perceptions when using the PROTEIN app is crucial, as it could shed light upon the role of specific structural characteristics and functionalities that further foster their intention to use the PROTEIN app for scaffolding their behavior change toward healthier living. Consequently, the PROTEIN app can have a positive footprint in people's nutritional and physical activity habits. In this vein, an experimental study was set up as a framework to acquire user's perspectives about the use of the PROTEIN app and model them, accordingly, as explained next.

### Study Design

A total of ninety-three adult participants in total were asked to download and use the PROTEIN app within a 2-month period (15 April -−15 June 2020) and then to participate in an online PROTEIN survey (refer to Supplementary Material – Online Survey), to give feedback about their use of the PROTEIN app. The type of sample design followed was the non-probability sampling design, using as sampling technique the convenience sampling, i.e., each respondent is selected for inclusion in the sample based on the ease of the access. Since the subjects must be smartphone users, the sample frame adopted was social media platforms/channels (e.g., Twitter, Instagram, Facebook), emails, and web advertisements, supported with flyers. During the registration process of the PROTEIN app, the participants were asked to fill in the required field and access the “Terms of Use” and “Privacy Policy” pages to complete the registration process, being, thus, informed about the purposes of the PROTEIN project and the processing of their personal data. Once registered, users were sent an email to activate their account and allow them access to the PROTEIN app.

The online PROTEIN survey was designed and developed by a multidisciplinary team within the PROTEIN Consortium, based on a “co-creation” process (86), in which nutrition and physical activity experts, researchers, end-users, designers, and developers explore a problem and produce solutions together, considering their different approaches, needs, and perspectives (87, 88). In this way, an open, active, and creative process, where all relevant stakeholders are engaged, was followed.

For the design of the online PROTEIN survey, the following requirements were considered: i) least time consuming, i.e., no more than 20 min for completing it; ii) simple, objective, and quantitative structure, i.e., as little free text as possible; iii) inclusion of demographic information; and iv) feedback acquisition on what is more/less appealing to users. After review rounds within the PROTEIN Consortium, the final version of the online PROTEIN survey resulted in an efficient version. The online survey consisted of 28 questions, divided into sections, including rating scale, multiple-choice, dichotomous, and open-ended questions (refer to Supplementary Material – Online Survey). The online PROTEIN survey was provided to users utilizing the SurveyMonkey platform (SVMK Inc., CA, USA).

From the 93 participants in the study, 85 (refer to full demographic characteristics in the Results section) provided online consent and fully completed the online PROTEIN survey; hence, only their responses were kept for the modeling analysis, described next.

### Proposed mTAM Framework and Related Hypotheses

**Figure 2:**
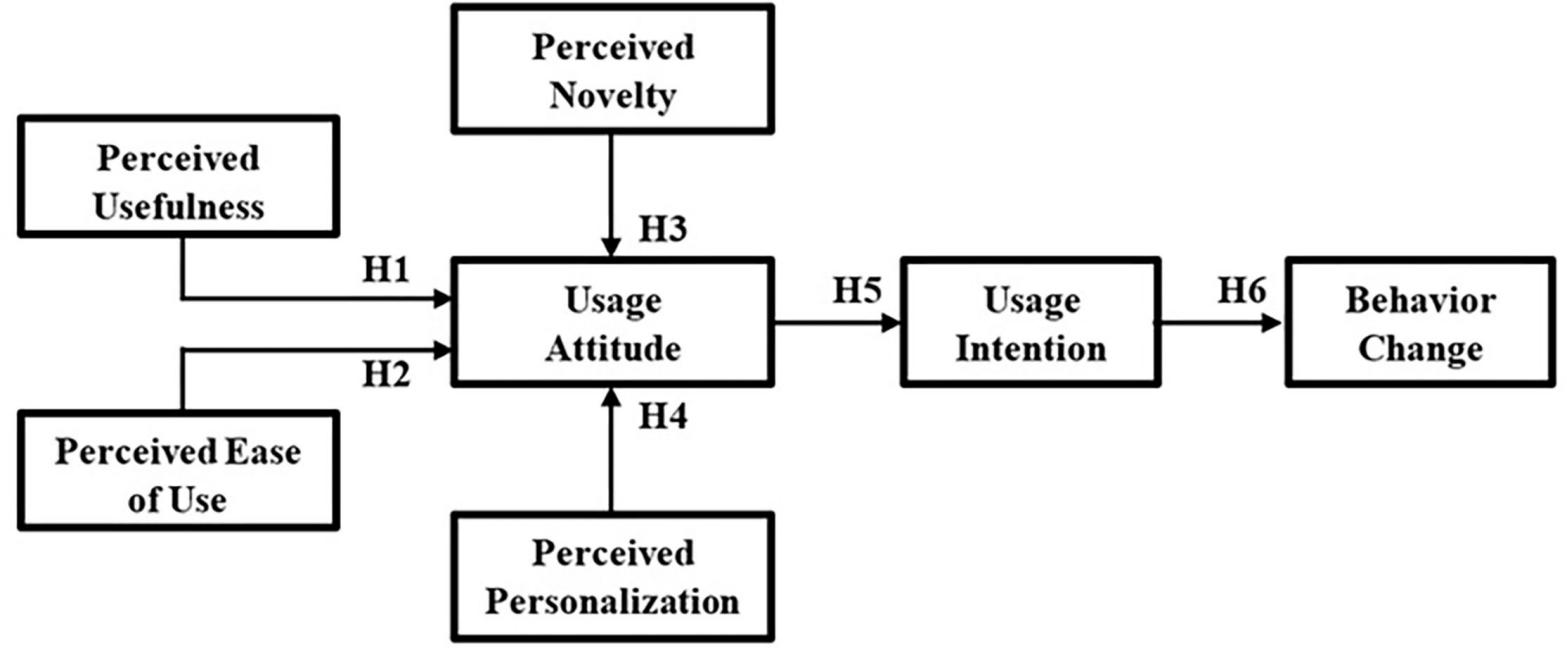
The proposed mTAM framework and the corresponding hypothesis (H1–H6).

Taking into consideration the TAM model (refer to theoretical background section), this study adopts the PU, PEoU, UA, and UI from TAM model and introduces two additional constructs, i.e., the “Perceived Novelty” (PN) and the “Perceived Personalization” (PP), toward the model output of “Behavior Change” (BC). This results in a modified TAM (mTAM) framework, as depicted in Figure 2.

To explore the contribution of each mTAM construct to the BC, a series of hypotheses is introduced. Davis et al. (39) mention that both PU and PEoU directly impact usage intention (UI) *via* the influence of UA. Moreover, the study of Holden and Karsh (89) states that the PU and the PEoU positively impact UA, showing that when users are confident that the adoption of novel technologies helps to improve their work performance, they exhibit more positive UA for adopting these novel technologies. In addition, if users perceive that a novel technology is easier to learn and that they do not need to apply much effort on the technology, then they have a more positive UA toward adopting this technology. This is also supported by the findings of Chopra et al. (90) that stress the influence of PU and PEoU to the UA, when evaluating the acceptance of dietary service applications by smartphone users using TAM. In the same vein, Mohammadi and Isanejad (91) found an influence of PEoU to UA, when evaluating the acceptance of IT in activity organization. From these perspectives, Hypotheses 1 and 2 are proposed below:

**Hypothesis 1** (H1): *Users' perceived usefulness toward the AI-based smartphone PROTEIN app positively influences usage attitude*.

**Hypothesis 2** (H2): *Users' perceived ease of use toward the AI-based smartphone PROTEIN app positively influences usage attitude*.

A crucial characteristic of any innovation is its novelty. Wells et al. (92) tried to frame the PN of an IT innovation as a salient affective belief in the nomological network related to the technology adoption. They conclude that the PN is an affective belief that plays a significant role in the adoption of IT innovations. So, if users perceive that novelty, it influences the adoption process, representing new alternatives to the existing ones, then they have a more positive UA toward adopting this technology. This was supported by the work of Robinson et al. (93), who found that new trends in technology engage the users and transfer them the notion that they use new products and technology before others, i.e., they become the leaders in acceptance of new ideas and products. In fact, novelty indirectly affects user's intentions and behavior, especially regarding new technological innovation (94). In this line, Hypothesis 3 is proposed as follows:

**Hypothesis 3** (H3): *Users' perceived novelty toward the AI-based smartphone PROTEIN app positively influences usage attitude*.

Personalized approaches seem to be needed to promote healthy behaviors. Personalization concepts associated with behavior change in mobile health can be categorized into four different dimensions, namely: users (related to user-related characteristics, e.g., personality, profile, need for cognition, and perception of social norms), system functionalities (related to the functionalities that can be found in apps, e.g., reminders, gamification features), information (related to the way information is transmitted, e.g., type of feedback provided), and mobile app properties (e.g., the aesthetics of the app) (95). In addition, a recent meta-analysis study that aimed to evaluate the impact of personalized mobile apps and fitness trackers on lifestyle behaviors (i.e., physical activity, diet, smoking and alcohol consumption) revealed that studies must explore the integration of multiple data from different sources and include personalized features other than content (96). In fact, smartphone users seem highly receptive to personalized content and services; personalized recommendations, for instance, can generate better responses according to the user personal preferences and interests (97). Some studies have also suggested that users' attitudes and behaviors can be affected by personalization aspects (98, 99). In this vein, it is very probable that users' PP affects UA toward UI and then to BC. Thus, we hypothesize the following (H4):

**Hypothesis 4** (H4): *Users' perceived personalization toward the AI-based smartphone PROTEIN app positively influences usage attitude*.

Davis et al. (39) and Robbins (100) state that UA directly influences UI, meaning that users' real usage behaviors are determined by UI, and that their UI are determined by their individual UA. In addition, Heberlein and Black (101) suggest that if UA and UI are more specific, then the relationship between the two is more distinct. From these perspectives, it becomes clear that if the users have a more positive UA toward a system, then they will have a higher UI toward its use. This is also supported by the work of Chen et al. (102), who explored the individual's attitude toward and intention to download and use dietary and fitness apps, and the findings of Okumus et al. (103), who explored the factors that influence customers' acceptance of smartphone diet apps when ordering food at restaurants. Hence, the Hypothesis 5 is proposed:

**Hypothesis 5** (H5): *Usage attitude of the users toward the AI-based smartphone PROTEIN app positively influences usage intention*.

Usage intention defines the degree to which an individual is willing to engage in a particular behavior (43). When individuals' UI is strong, the probability that they will engage in that behavior is higher, and consequently, UI and real behaviors present a strong correlation. Furthermore, UI has been considered the best variable to predict an individual's behavior (43). This view is also adopted in the work of West et al. (104), who analyzed how diet and nutrition-related mobile apps lead to behavior change, and in the work of Requero et al. (105), where the enhancement of the correspondence between attitudes and behavioral intentions was explored as promoter of healthy eating. In this vein, Hypothesis 6 is proposed, as follows:

**Hypothesis 6** (H6): *Usage intention of the users toward the AI-based smartphone PROTEIN app positively influences behavior change*.

### Statistical Analysis

Stata version 14 (StataCorp) was used for the statistical analysis. Descriptive statistics were calculated for demographics and the related questions from the online survey (refer to Supplementary Material – Online Survey). To explore the magnitude of the influence between the mTAM constructs, a linear regression analysis was adopted and the corresponding parameters, i.e., coefficient of determination (R^2^), adjusted R^2^ (adj R^2^= 1 - $\left[\frac{\left(1-R^{2}\right)\left(N-1\right)}{N-k-1}\right]\leq \;R^{2}$, where N is the number of points in the data sample and k is the number of independent regressors (variables), excluding the constant), regression coefficient with standard error (SE), t ratio (the estimate divided by the SE), p - value, and 95% confidence interval (CI), were estimated. Values of *p* < 0.05 and |*t*| > 1.96 were set as thresholds for adopting statistically significantly difference from 0 of the coefficient's estimate at the 95% CI, hence for adopting the corresponding hypothesis. Moreover, the F-statistics, i.e., the ratio of the mean sum of squares of the model to that of the residual, were also estimated for measuring how the ratio of the explainable mean variance to the unexplainable mean variance is statistically >1. The corresponding degrees of freedom for the model and residual are given in parentheses.

The regression analysis has been selected instead of the structural equation modeling (SEM) (106–110), as the latter represents and relies upon the causal assumptions of the researcher, and its credibility depends on the credibility of the causal assumptions in each application (111). Moreover, there is a semantic difference between the coefficients originating with a regression where no causal assumptions are made vs. from a SEM that makes strong and weak causal assumption (111). In this way, with the adopted regression analysis, any bias from the classification to strong and weak causal assumptions in the construction of the mTAM framework is avoided.

The reliability and validity of the items used in the proposed mTAM framework have been tested, accordingly. In particular, for the reliability analysis to examine the internal consistency and stability of grouped variables, denoting how closely related a set of items are as a group, the Cronbach's α was estimated as a $=\frac{M\bar{C}}{\bar{var}+\left(M-1\right)\bar{C}},$ where M is the number of grouped items, $\bar{C}\;and\;\bar{var}$ are the average covariance between item - pairs and average variance, respectively. Values of a ≥ 0.70 denote acceptable internal consistency (112). With regard to the validity analysis, the confirmatory factor analysis (CFA) (113) was adopted, and the factor loading (FL) with the related *p*-value, average variance extracted (AVE), and composite reliability (CR) parameters were estimated. According to Nunnally (114), if FL > 0.5, AVE > 0.5, and CR > 0.7, then the research variables exhibit good convergent validity.

## Results

### Demographic Characteristics

The PROTEIN app was evaluated by eighty-five (*n* = 85) volunteers coming from eight European countries (i.e., Greece, Portugal, Germany, UK, Belgium, Italy, the Netherlands, and Serbia) with average age of 49.3 years (±*std* = 16.3 *years*; *age range* = [25−44] *years*), who responded anonymously to the online survey (average missing values <4). The highest percentage of the respondents, 32%, were in the 35–44 age groups followed by 30% of 25–34 years old, then 16% of 18–24 years old, 12.5% of 44–54, 7% of 55–64, and the least, 2.5% was for 65 and over years old. Moreover, the distribution of male/female was 56% male and 41.5% female. In terms of body weight and body height, the means were 72.5 kg (±*std* = 16.5 *kg*) and 164 cm (±*std* = 8.4 *cm*). The majority of users (74%) were classified as general public and therefore did not belong to any clinically defined group (such as users under medical/nutrition supervision).

### General Users' Perspectives

The survey responses of the PROTEIN app users provided some insights into their general opinions of the app. In particular:

1. Primary usage goal: the most important primary health/well-being goal pointed out by the respondents were “Be more active,” “Eat more healthily,” and “Lose weight,” revealing that good nutrition, physical activity, and a healthy body weight are the essential components of a person's overall health and well-being. On the opposite side, the following statements: “Manage an existing health condition,” and “Manage specific dietary requirements” were considered by the respondents as the least important primary health and well-being goal.
2. Usefulness: this was perceived by the users as the ability of PROTEIN app to improve health, encourage more exercise, and provide suitable recommendations for activities and healthier meal plans.
3. Ease of use: this was perceived by the users *via* the easiness of interacting with and organizing the supermarket shopping list, fostering the easy customization of personalized plans with wide variety and alternative options within the meal plan and activities (number and types of suggested meals and activities), or fast and easy-to-use functionalities of adding a meal and/or an activity.
4. Personalization: adaptation to the personalized characteristics of each user was considered important in the user profile of the PROTEIN app, covering many important attributes related to potential allergies, insufficiencies, intolerances, or personal diet. In addition, the personalized feedback, in terms of personalized notifications and achievements, contributed to boosting the user perception of PROTEIN app personalization feature.
5. Novelty: the users expressed their perception of novelty *via* the uniqueness and variety of the features provided by the PROTEIN app, compared to the other apps that relate to nutrition and physical activity, especially mentioning the feature of recording the meal/activity.

The aforementioned perspectives are propagated through the usage attitude and intention as means for activity and eating behavior change, as reflected in the adopted mTAM and the related hypothesis testing results as presented below.

### Hypotheses Testing

**Figure 3:**
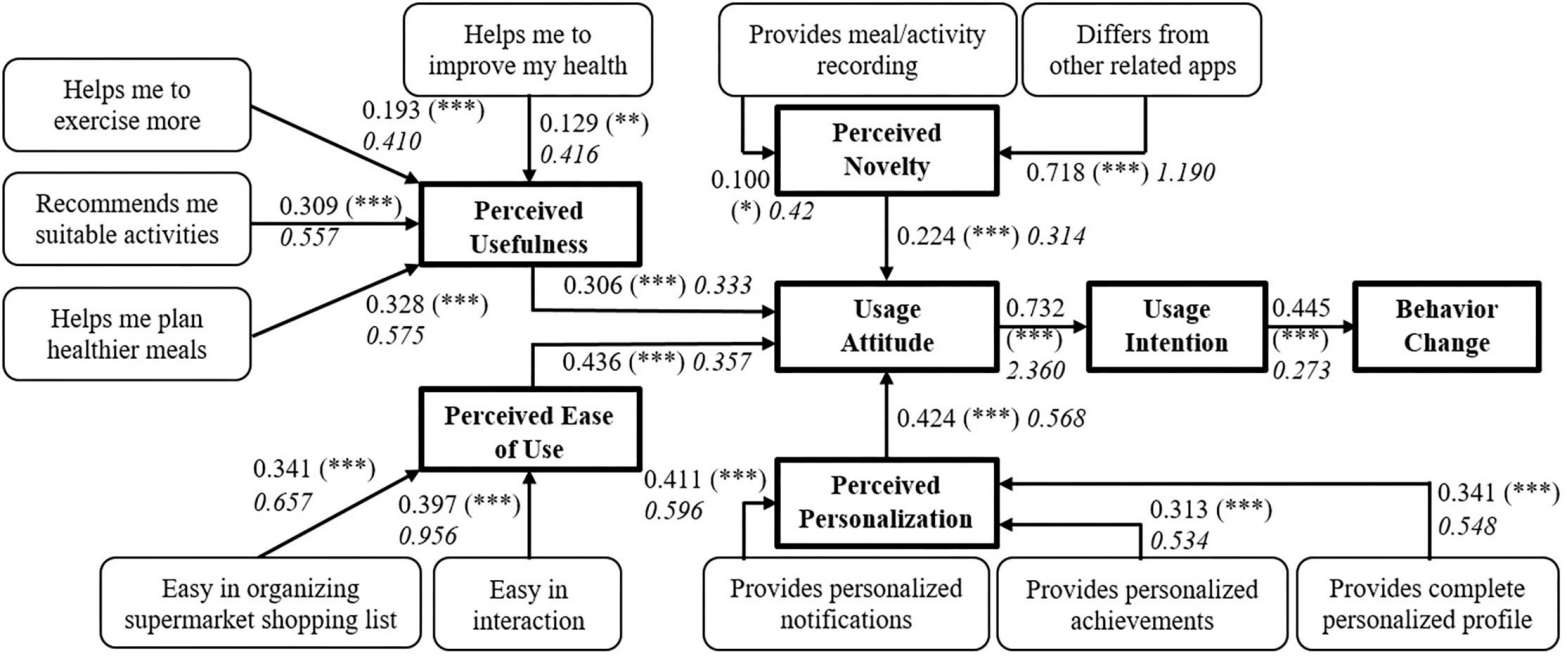
Path relationships between mTAM main constructs (Figure 2) and related items via the values of adj *R*^2^ and the estimated regression coefficients (in italics). *p < 0.05; **p < 0.01; ***p < 0.001.

Figure 3 depicts the path relationships between mTAM constructs (Figure 2) and the related items *via* the values of adj R^2^ (^*^ p < 0.05;^**^ p < 0.01; ^***^ p < 0.001), as they were derived from the performed regression analysis. Moreover, Table 1 tabulates all the corresponding results regarding different regression paths that relate items with separate constructs (nos. 19), and the regression paths between constructs that support the hypothesis testing outcome (nos. 1015), along with the corresponding Cronbach's α (where applicable). The effect of sex, age range, and BMI was tested for all the regression paths tabulated in Table 1. From this analysis, statistically significant differences related to sex were found for the regression paths of nos. 14 (p = 0.028, 0.013, 0.019, and 0.017, respectively). Moreover, statistically significant differences related to age range and BMI were only found for the regression paths of no 12 (p = 0.041) and no 13 (p = 0.002), respectively.

**Table 1:** Reliability, validity and least squares regression analysis results for the adopted mTAM model (Figures 2, 3).

**Regression path**			**Reliability Analysis**	**Validity Analysis**			**Hypothesis (AC/RJ)**	**R^2^**	**adj R^2^**	**Coefficient (SE)**	**F (df_**M**_,df_**R**_)**	**t**	**p**	**95% CI**
**No**	**Independent variable(s)**	**Dependent variable**	**Cronbach's a**	**FL**	**AVE**	**CR**								
1	“Helps me to improve my health”	PU	0.8367	0.72 (***)	0.531	0.815	-	0.145	0.129	0.416 (0.137)	9.18 (1.54)	3.03	0.004	[0.141:0.692]
2	“Helps me to exercise more”			0.90 (***)			-	0.207	0.193	0.410 (0.106)	14.91 (1.57)	3.86	***	[0.197:0.624]
3	“Recommends me suitable activities”			0.65 (***)			-	0.322	0.309	0.557 (0.108)	26.13 (1.55)	5.11	***	[0.338:0.775]
4	“Helps me plan healthier meals”			0.60 (***)			-	0.328	0.315	0.575 (0.111)	26.84 (1.55)	5.18	***	[0.352:0.798]
5	“Easy in organizing supermarket shopping list”	PEoU	0.7757	0.45 (***)	0.581	0.710	-	0.341	0.327	0.657 (0.133)	24.40 (1.47)	4.94	***	[0.389:0.924]
6	“Easy in interaction”			0.98 (***)			-	0.397	0.387	0.956 (0.155)	37.63 (1.57)	6.13	***	[0.644:1.268]
5	“Provides novel meal/activity recording”	PN	0.7674	0.48 (***)	0.585	0.717	-	0.110	0.100	0.42 (0.165)	6.44 (1.52)	2.54	0.014	[0.088:0.753]
6	“Differs from other related app”			0.97 (***)			-	0.724	0.719	1.190 (0.102)	134 (1.51)	11.58	***	[0.984:1.397]
7	“Provides personalized notifications”	PP	0.8071	0.93 (***)	0.515	0.735	-	0.421	0.411	0.596 (0.092)	41.53 (1.57)	6.44	***	[0.411:0.782]
8	“Provides personalized achievements”			0.76 (***)			-	0.352	0.313	0.534 (0.102)	27.47 (1.57)	5.24	***	[0.330:0.739]
9	“Provides complete personalized profile”			0.32 (***)			-	0.353	0.341	0.548 (0.097)	31.64 (1.58)	5.62	***	[0.353:0.743]
10	PU	UA	0.8456	0.77 (***)	0.541	0.821	H1 (AC)	0.318	0.306	0.333 (0.065)	26.12 (1.56)	5.11	***	[0.202:0.463]
11	PEoU			0.91 (***)			H2 (AC)	0.446	0.436	0.357 (0.053)	45.07 (1.56)	6.71	***	[0.250:0.463]
12	PN			0.59 (***)			H3 (AC)	0.239	0.224	0.314 (0.078)	16.06 (1.51)	4.01	***	[0.156:0.471]
13	PP			0.63 (***)			H4 (AC)	0.434	0.424	0.568 (0.085)	43.81 (1.57)	6.62	***	[0.396:0.740]
14	UA	UI	-	0.86 (***)	-	-	H5 (AC)	0.740	0.732	2.360 (0.188)	156.90 (1.56)	12.53	***	[1.982:2.737]
15	UI	BC	-	0.67 (***)	-	-	H6 (AC)	0.455	0.445	0.273 (0.040)	46.80 (1.56)	6.84	***	[0.193:0.353]

FL, Factor Loading; AVE, Average Variance Extracted; CR, Composite Reliability; AC, Accepted; RJ, Rejected; adj, Adjusted coefficient of determination R^2^; SE, Standard Error; F(df_M_,df_R_), F-statistics with df_M_ and df_R_ corresponding to the degrees of freedom for the model and the residual, respectively; CI, Confidence Interval; PU, Perceived Usefulness; PEoU, Perceived Ease of Use; PN, Perceived Novelty; PP, Perceived Personalization; UA, Usage Attitude; UI, Usage Intention; BC, Behavior Change; ^***^ p < 0.001.

From a general perspective of the results presented in Table 1, it can be shown that the Cronbach's α values range from 0.7674 (group of items nos. 5, 6) up to 0.8456 (group of constructs nos. 10–13), revealing that the grouped items and constructs all have good internal consistency and reliability. In addition, the corresponding validity results, also tabulated in Table 1, show that all items have FL > 0.5, CR > 0.7, AVE > 0.5, indicating that the items/constructs within the mTAM framework have good convergent validity. All estimated values of F-statistics were significantly >1 supporting the acceptance of the related hypothesis. Moreover, in almost all cases, the estimated p - values are less than 0.001, with the exception of the regression paths no. 1 (p <0.01) and no. 5 (p <0.05), indicating extreme statistical significance. Additionally, the t ratio ranges from 2.54 (no. 5) up to 12.53 (no. 14), justifying the statistical validity of the estimated regression coefficients. Furthermore, R^2^ varies from 0.110 (no. 5) up to 0.740 (no. 14) and the corresponding adj R^2^ ranges from 0.100 (no. 5) up to 0.732 (no. 14), showing that, in most cases, there is a good fit of the regression models used to express the relation between the items/constructs of the proposed mTAM framework.

Following the paths of Figure 3, combined with the results of Table 1, it can be seen that the “Helps me to improve my health”, “Helps me to exercise more,” “Recommends me suitable activities,” and “Helps me plan healthier meals” items collectively contribute to the PU construct (Cronbach's α = 0.8367) of the mTAM. The corresponding adj R^2^ values are 0.129, 0.193, 0.309, and 0.328, respectively, showing that the plan of healthier meals explains the highest part of the PU variance, compared to the “Recommends me suitable activities,” “Helps me to exercise more,” and “Helps me to improve my health” items. Moreover, the “Easy to organize supermarket shopping list” and “Easy in interaction” items both contribute to the PEoU construct (Cronbach's α= 0.7757) of the mTAM. The corresponding adj R^2^ values are 0.327 and 0.387, respectively, revealing almost an equal contribution in the explanation of the PEoU variance. In its turn, the “Provides novel meal/activity recording” and “Differs from other related app” items collectively contribute for the PN construct of the mTAM α (Cronbach's α = 0.7674). Clearly, the latter item exhibits much higher adj R^2^ value (0.719) when compared to the one from the former (0.100). This is anticipated, as the concept of novelty is first conveyed to the users via the comparison of the PROTEIN app with the state - of - the - art and then via the feature of the meal/activity recording. For the PP construct of the mTAM, the “Provides personalized notifications,” “Provides personalized achievements,” and “Provides complete personalized profile” items collectively contribute (Cronbach's α = 0.8071). From these items, the “Provides personalized notifications” item exhibits the highest adj R^2^ of 0.411, followed by the “Provides complete personalized profile” (adj R^2^= 0.341) and the “Provides personalized achievements” (adj R^2^ = 0.313) items. This shows that the sense of personalization is perceived by the users textitvia personalized feedback and the ability to customize the user's profile, taking into consideration the specificity of each user's preferences and sensitivities (e.g., allergies, insufficiencies).

Moving on to the hypotheses testing, the results from Table 1 justify that all hypotheses, i.e., H1-H6 (refer to Figure 2) can be accepted. In particular, there is a collective contribution of the PU, PEOU, PN, and PP constructs to the UA construct (Cronbach's α = 0.8456). In addition, the corresponding adj R^2^ values are 0.306, 0.436, 0.224, and 0.424, respectively, showing that the PEoU explains a high part of the variation in the UA, followed by the PP, PU, and PN. There is a strong connection between the UA and UI constructs, as the corresponding adj R^2^ value is 0.732, revealing that the establishment of a solid usage attitude will effectively lead to an established usage intention. Consequently, this solidification could feed the BC construct (adj R^2^ = 0.445), explaining a significant part of its variance.

The aforementioned results showcase the validity of the proposed mTAM framework and related hypotheses to explain the interaction between the items and constructs that are activated during the process of PROTEIN app evaluation and its potential users' acceptance.

## Discussion

Here, we present the use of the mTAM framework as a means to model the users' evaluation of the PROTEIN app, a smartphone-based nutrition and activity app developed by a multidisciplinary team to support self-management of dietary intake and physical activity and scaffold any necessary behavior change that would lead to healthier living. In view of this approach, the six hypotheses, i.e., H1–H6, of the proposed mTAM framework provide a fine-grained breakdown of the elements that are gradually built during the user's interaction with the PROTEIN app, explaining the dependencies and power of these constructs to provide a meaningful explanation of the users' perspectives on usefulness, easiness, novelty, personalization, usage attitude, and usage intention toward behavior change.

Overall, nutrition and physical activity-related mobile apps show promise as tools to successfully facilitate positive health behavior change. Furthermore, mobile apps that focus on improving motivation, feedback, self-efficacy, attitudes, knowledge, and goal setting may be particularly useful (104), as in the case of the PROTEIN app. Meal planning including meal variety, in particular, is considered one of the nutrition counseling strategies that facilitate food behavior changes (115). Moreover, meal planning can be viewed as one technique to deliver nutrition knowledge in a more practical way. When expert-verified knowledge is embedded within the related knowledge-based systems, personalization could be enabled in providing personalized feedback about healthy lifestyle, complying, at the same time, with established and ethical guidelines of different fields of nutrition research.

Based on the Methontology, i.e., a methodology for building ontologies (116), the PROTEIN app has included the nutrition and activity (NAct) ontology. The latter was created based on experts' knowledge from the fields of nutrition, activity, and health fields. In NAct ontology, each subject's implicit/explicit goals related to nutrition and well-being are connected with his/her situational condition and standardized European nutritional and well-being directives (117). Overall, the NAct ontology models: i) in a slim and holistic manner food-specific nutritional information and activity-specific well-being information; ii) nutritional and well-being user goals and relates them with nutritional and well-being information; iii) medical conditions, allergies, intolerances, deficiencies, and lifestyle dietary choices and relates them with nutritional and well-being information; and iv) properties that define specificities of the aforementioned relationships that aid in the selection of appropriate meals and physical activities for a given person. Adopting a holistic approach toward behavioral change perspectives, the NAct ontology has been integrated with the PROTEIN app AI advisor, which employs the LiFR fuzzy reasoning (118) scheme. In this way, the PROTEIN app infers the optimal recommendations for meals, restaurant menu items, and physical activities to each user, taking into consideration his/her dietary and medical profile.

The aforementioned design of the PROTEIN app, which combines NAct ontology with LiFR, contributes to the constructs of the adopted mTAM framework. Clearly, the users' evaluation results showcase the various capabilities of the PROTEIN app to facilitate meal and physical activity planning, to reflect about and create appropriate shopping lists, to be aware of the impact of eating healthy food and be more active, to easily interact with and find it as a useful tool, to have valuable personalized feedback and profile, taking into consideration specific nutrition (e.g., allergies, intolerances) or physical activity (e.g., herniated disk) issues. All these contribute to the users' UA of the PROTEIN app (H1–H4) and support UI to adopt it as a means (H5) that could potentially lead to their behavior change (H6), in terms of clarifying/improving nutritional and physical activity beliefs/habits. To further examine the mediating role of UA and UI in the BC, we performed hierarchical linear regression, examining the prediction of BC from UA and the prediction of BC from UA considering as mediator the UI. The results of this analysis are tabulated in Table 2. As it is seen from the latter, there is a significant change in the prediction of BC from UA when the UI is used as a mediating variable, since the regression coefficient in Path No. 1 (refer to Table 2), i.e., 0.640 (*p* < 0.001), is reduced to−0.071 (*p* = 0.742) when moving to Path No. 2. This is further justified by the difference in the *R*^2^ and adj *R*^2^ between the Path No. 1 and Path No. 2 (Table 2), which are statistically significant, since the corresponding estimated *p*-value equals to 0.001. On the contrary, the prediction of BC from UI when considering the UA is not severely affected, since the estimated regression coefficient (0.295; *p* < 0.001) does not significantly deviate from the estimated value in the regression Path No. 15 (Table 1), i.e., 0.273 (*p* < 0.001).

**Table 2:** Hierarchical regression results for examining the mediation role of UA, UI in BC prediction in the adopted mTAM model (Figures 2, 3).

**Hierarchical Regression path**				**R^2^**	**adj R^2^**	**Coefficient (SE)**	**F (df_**M**_, df_**R**_)**	**t**	**p**	**95% CI**
**No**	**Independent variable**	**Mediating variable**	**Dependent variable**							
1	UA	-	BC	0.322	0.310	0.640 (0.122)	27.16 (1.57)	5.21	***	[0.394:0.886]
2	UA			0.456	0.436	−0.071 (0.215)	23.09 (2.,55)	−0.33	0.742	[−0.504:0.361]
		UI				0.295 (0.078)		3.77	***	[0.138:0.452]
Mediation effect of UI (Path No 1 vs. Path No 2)				*R^2^* diff= 0.134	adj *R^2^* diff=0.126		12.385 (1.55)		0.001	

Adj, Adjusted coefficient of determination R^2^; SE, Standard Error; F(df_M_,df_R_): F-statistics with df_M_ and df_R_ corresponding to the degrees of freedom for the model and the residual, respectively; CI, Confidence Interval; UA, Usage Attitude; UI, Usage Intention; BC, Behavior Change, ^***^ p < 0.001.

The role of the mobile apps as moderators for changing diet or physical activity behaviors/habits has been explored in various clinical settings (13, 119–122). In particular, the work of Spring et al. (119), through a 12-month randomized controlled trial (RCT) involving 70 adults (BMI∈[35−40] *kg*/*m*^2^), revealed that users with a personal digital assistant (PDA) to input a description of their daily meals and self-regulate their energy intake *via* a “goal feedback thermometer” updated with their current caloric intake, achieved greater weight loss than their control group counterparts. In another 24-month RCT, namely, Self-Monitoring and Recording Using Technology (SMART) (120), involving 210 adults with overweight/obesity, it has shown that the use of PDA with appropriate feedback contributed to the adults' dietary behavior change, resulting in significant weight loss when compared to those using paper diaries only for weight loss and maintenance monitoring. A 3-month RCT, namely, DialBetics (121), highlighted the benefit of using a smartphone-based app for self-managing type 2 diabetes with better control of the nutritional related parameters, i.e., the HbA1c and fasting glucose, when compared to the control group users. In a 10-week RCT described by Zhou et al. (122), the use of a smartphone app (incorporating machine learning) for delivering personalized information for step goals and physical activity monitoring was more effective in keeping the users more active than the allocation of a preset step goal per day. This shows the effect of the app personalization feature in achieving and maintaining active living. In a 9-month mPED RCT reported in (13), including 210 community-dwelling physically inactive women, the use of a smartphone app that fed back to the intervention group users their steps and physical activity resulted in increased physical activity when compared to the control group ones in the first 3 months and allowed them to maintain their physical activity for the next 6 months.

The aforementioned works highlight the efficiency of the related apps to be incorporated in clinical settings and act as the mediators for scaffolding the users' efforts toward healthier living. Actually, incorporation of mobile apps in the design of health promotion programs is growing, following the profound mobile app market exponential growth (123). Nevertheless, careful evaluation of the underlying “science” of such mobile apps is needed, to ensure that their characteristics and functionalities really reflect evidence-/theory-based principles of behavior change, maximizing, and their effectiveness (12, 124, 125). Moreover, the actual use (especially sustained use) of mobile apps to track health and related behaviors is questionable (126), especially when the user is overwhelmed and saturated by the manual tasks that s/he has to undertake, to successfully interact with the mobile app. A recent systematic review (13) explored the role of the mobile apps in the improvement in health behaviors and outcomes, such as physical activity, nutrition, drug, alcohol use, and mental health, showcasing their effectiveness as health behavior change moderators. The findings highlight the lack of appropriate framework(s) that accurately evaluate the role of the mobile health app in effectively promoting behavior change, hence affecting the evidence in supporting their key role in this behavior change (13). This finding, however, enhances the contribution of the proposed mTAM framework as a means for explaining the key constructs that could most contribute to shaping and supporting such health behavior change. Taking into consideration the validation of the hypotheses H1–H6 in the mTAM framework, the focus on various constructs could shed more light on the necessary features that the related nutrition and physical activity app should have, maximizing their contribution to the achievement of such constructs. Apparently, the PROTEIN app includes a variety of such features that makes it appealing to the users, with high perceived novelty (adj *R*^2^ = 0.718), showing adequate acceptance and intention to be used in self-managing and supporting healthy eating and physical activity.

As expressed *via* the identified interdependencies between the mTAM items/constructs (Figure 3, Tables 1, 2), the users appreciate various perspectives of interaction with the PROTEIN app, i.e., assistive, informative, adaptive, personalized, novel, and rewarding, to build their attitude and intention of usage toward behavior change. From the users' perspective, these results reveal also some insights into understanding more about how they link themselves with the PROTEIN app. In particular, it seems that the establishment of the sense of instant assistance and/or real-time reliable information acquisition about their nutrition and activity in a personalized way positively affects their receptiveness to different PROTEIN app features and/or interaction strategies. PROTEIN app adaptivity and personalization using its AI-based engine can absorb any predisposition of the user to benefit from its use or put them at risk for negative consequences by influencing what type of information/features one attends to, how one processes that information, and subsequently uses the information/features to self-regulate own behavior (127, 128). There are limited data on the associations between mHealth app use and psychological characteristics of its users (126), and the demographic characteristics of the participants of this study, along with the online survey focus, do not support exploration of such dimension here. However, some works have linked different aspects of personality to different features of persuasive technologies (129, 130) and social cognitions (e.g., barriers, attitudes, and behavioral control) being predictive of technology uptake outcomes [e.g., intention to (131) or intensity of (132) mHealth app use], pointing to some potential dimensions for further incorporation within the mTAM framework.

From an implications' perspective, our findings provide actionable insights. In particular, the involved constructs in the mTAM framework highlight the need for: a) clear understanding of the users' health needs and introduction of apps with high degree of novelty, compared to the ones currently in the market, personalization, adaptation capabilities, and scalability within the growing market to meet different users' needs; the use of AI (as in the PROTEIN app) can contribute toward such need; b) easy-to-use apps, since the individual's decision to actually perform a health behavior has to be acted on in efficient and quick way (133). Consequently, the simplification in the registration and in the means of data acquisition provides a clear reduction in the user's effort when interacting with the app, and c) apps with clear goal setting, self-monitoring functionalities, personalized feedback, and instructions, as a means of establishing and retaining users to establish and extent users' retention, sustaining a balanced interaction flow between challenges and skills, helping the users in setting challenging but achievable tasks as goals to pursue.

Despite the promising results, some limitations of this work should be highlighted. Clearly, the number of participants (*n* = 85) may not be considered adequate for generalization of the findings to larger populations. However, as the average age of the participants is around 50 years, coming from eight European countries, we could argue that the sample is representative enough to ground the proposed mTAM model, assuming that, as adults with living experience, their understanding of the PROTEIN app use and the related survey may be adequate. Extending the sampling to larger and more diverse populations could further justify the validity of the current findings. The PROTEIN consortium has already scheduled the testing of the proposed mTAM framework in a second round of pilot studies that will involve a higher number of participants, an updated version of the PROTEIN app to include also iOS smartphone users, and populations, apart from the healthy ones, with specific characteristics, e.g., patients with diabetes, patients with cardiovascular disease, patients with obesity/overweight, and vulnerable population. The users' time period of the PROTEIN app use (2 months) sets also another limitation. In general, meeting the expectation for the healthier lifestyle goal requires long-term effort, and immediate rewards usually do not appear as substantial positive outcomes. However, the proposed mTAM framework provides the opportunity for a dynamic perception of its constructs, using it in a time-scale manner. In fact, mTAM outcomes could be evaluated upon meeting intermediate (short/mid-term) goals, providing output for the constructs that strongly participate in the sustainability of the users' positive perspective of the app, hence keeping them using the app. In this context, time-scaled versions of the mTAM could be combined to provide an explanation of the time-based dynamics of users' continuance intention (CI) for health apps (134–136). This would shed light upon the factors that can explain why many users discontinue to use the health app after its initial adoption. Thus, the mTAM model at specific time scale(s) could be enriched with additional constructs that may focus at explaining continued use or abandonment. For instance, some social, e.g., subjective norms (136) and psychological factors, e.g., health consciousness and flow (136–138) have been proposed to explain some technology usage patterns. A clear example of such factors is the disruptive effect of the COVID-19 pandemic on many people's health routines and status. The restriction on outdoors activities shifted the work out space from the gym to home. This has resulted in a download spike of health and fitness apps, combined with home work out equipment sales (139). Moreover, a spike in food delivery and online shopping due to COVID-19 altered the way nutrition is perceived (140). Considering that the PROTEIN app was evaluated during the core COVID-19 pandemic period, its functionality to assist the users in organizing their supermarket list (adj *R*^2^ = 0.397) showed its capabilities to support healthy online food ecosystem.

In conclusion, this study provides evidence for the efficiency of the mTAM framework to express the users' perspectives about the PROTEIN app, an AI-based smartphone app that provides personalized recommendations for self-managing food intake and physical activity. The six hypotheses examined showcase the importance of different mTAM constructs to promote the behavior change in the users toward healthier living, by improving their eating and physical activity habits. In addition, the mediating role of the usage intention to the behavior change potential was explored, recommending focus upon the way the intention of using the PROTEIN app is scaffolded by its actual structural characteristics. The proposed mTAM framework can be useful in constructing recommendations for the relevant apps' design, revealing which aspects, in terms of structure, user interface, functionality, way of interaction, personalization, are considered important by the users to lead them in behavior change toward healthier living. Further evaluation studies are warranted to validate the power and generalization of the proposed mTAM framework, employing its time-scaled versions for also exploring the users' continuance intention.

## Data Availability Statement

All data generated and analyzed during the current study are available from the corresponding author on a reasonable request.

## Ethics Statement

The studies involving human participants were reviewed and approved by Local Ethics Committees and the corresponding IRB approvals were obtained for the research (Lisbon, Portugal CEIFMH 14/2021; Thessaloniki, Greece 2/20.09.2021; Berlin, Germany EA4-110-20). Online informed consent was obtained from every participant prior to enrolment in the online PROTEIN survey. The patients/participants provided their written informed consent to participate in this study.

## Author Contributions

SD: conceptualization, study design, organization, and writing the manuscript. YO: online PROTEIN survey realization in the SurveyMonkey platform. LH: conceptualization, study design, organization, statistical analysis, manuscript review, and update. All authors contributed to the conceptual design and development of the PROTEIN mobile application and online PROTEIN survey, discussed the results and reviewed the manuscript, have read, and agreed to the published version of this manuscript.

## Funding

The research leading to these results has received funding from the European Union's Horizon 2020 Research and Innovation Programme under grant agreement no. 817732 (PROTEIN: PeRsOnalized nutriTion for hEalthy living).

## Conflict of Interest

CV was employed by Fluviale SRL. YO and AB were employed by the company Intrasoft International SA. SG was employed by the company Datawizard. GT was employed by the company PLUX. JB was employed by the company Grupo CMC. DR and JK were employed by the company OCADO. SM was employed by the company AgriFood Capital BV. PB was employed by the company Healthium/Nutrium. The remaining authors declare that the research was conducted in the absence of any commercial or financial relationships that could be construed as a potential conflict of interest.

## Publisher's Note

All claims expressed in this article are solely those of the authors and do not necessarily represent those of their affiliated organizations, or those of the publisher, the editors and the reviewers. Any product that may be evaluated in this article, or claim that may be made by its manufacturer, is not guaranteed or endorsed by the publisher.

## Footnotes

^1^ https://buff.ly/2Qz3M7i
