# Supplementary material for: Users' Perspective on the AI-Based Smartphone PROTEIN App for Personalized Nutrition and Healthy Living: A Modified Technology Acceptance Model (mTAM) Approach
Source: Front Nutr. 2022 Jul 1;9:898031. doi: 10.3389/fnut.2022.898031 (PMC9307489; doi:10.3389/fnut.2022.898031)
Supplement: Supplementary file 1 [file Data_Sheet_1.PDF]

PROTEIN

PERSONALISED NUTRITION FOR HEALTHY LIVING

PROTEIN H2020 Questionnaire

1. Welcome

This project has received funding from the European Union's Horizon 2020 research and innovation programme under grant agreement №817732

This is an anonymous survey. No personal information will be collected. You will not be requested to disclose any personal information. The researchers will not be able to identify an individual respondent. IP Logging is disabled.

Data collected from the questionnaires through the survey will be kept for the specific duration of the research period and will only be used for the purposes of the PROTEIN Project (H2020 GA No817732). After that period, the data will be destroyed in line with the project's contractual obligations to the European Commission. Access to the data is exclusively allowed to authorized members of the PROTEIN consortium, engaged in the aforementioned research activities.

Participation in the following online survey is voluntary.
Thank you for participating. Your feedback is important.

* 1. **I agree with the Terms of this survey (see disclaimer below)**,
including its objectives, content and tool, and I wish to participate.

- ◯ Yes! I consent. Let's go
- ◯ No! I do not consent

DISCLAIMER

PROTEIN (“PeRsOnalized nutriTion for hEalthy livINg”; Grant agreement ID: 817732; https://cordis.europa.eu/project/id/817732), co-funded under the European Union Framework Programme for Research and Innovation ‘Horizon 2020’.

PROTEIN is a research and innovation project which aims to promote a healthy lifestyle and help improve the general state of health of the European population by combining the latest technologies to offer personalized nutrition and physical activity plans.

The project runs from 1 December 2018 to 31 May 2022. The study director for the present survey is INTRASOFT International S.A. For detailed information regarding PROTEIN, its consortium, and its activities, you can visit the project´s website and social media.

Participation in the following online survey is purely voluntary, based on your informed consent. Your responses to the survey will be aggregated with the responses of other users, in order to produce statistical information, necessary for the purpose of the determination of end-user requirements, the improvement of the PROTEIN app, and the drafting of the respective project deliverables. The survey consists of 26 questions and will take around 15-20 minutes to be filled in. Each section includes free text fields and multiple choices.

Please fill in the survey anonymously. You will not be requested to disclose any personal information. The researchers will not be able to identify an individual respondent. Thus, when drafting your responses, please abstain from including any information relating to you or any other identified or identifiable person (names, email addresses, phone numbers, etc.). For updates, you can follow the progress of the research on the project's website and its communication channels.

Data collected from the questionnaires through the survey will be kept for the specific duration of the research period and will only be used for the aforementioned purposes. After that period, the data will be destroyed in line with the project’s contractual obligations to the European Commission. Access to the data is exclusively allowed to authorized members of the PROTEIN consortium, engaged in the aforementioned research activities.

PROTEIN uses a third-party platform (SurveyMonkey) for the creation and conduct of this survey. This platform is developed and maintained by SVMK Inc., One Curiosity Way, CA 94403, San Mateo, USA. The PROTEIN researchers use the anonymous function of the survey tool. Before proceeding with the questionnaire, please read the SurveyMonkey Privacy Policy.

For more information regarding the survey and the project please contact Lazaros Gymnopoulos at lazg@iti.gr. Questions relating to the processing of personal data can be directed to Olga Gkotsopoulou at olga.gkotsopoulou@vub.be. If you consider that your rights under the General Data Protection Regulation have been infringed as a result of the processing of personal data, you are also entitled to submit a complaint with a National Supervisory Authority.

By clicking "I consent" with the Terms, you freely consent to take part in this survey and you confirm that you have read and fully understood the above statement.

1 / 10 10%

Next

Powered by
SurveyMonkey
See how easy it is to create a survey.

PROTEIN H2020 Questionnaire

2. Information

* 2. Please indicate your age

◯ 18-24
◯ 25-34
◯ 35-44
◯ 45-54
◯ 55-64
◯ 65 and over

* 3. Please indicate your gender

◯ Male
◯ Female
◯ Rather not say

4. Please indicate your weight (in kgs)

0 — 150

5. Please indicate your height (in cm)

0 — 250

2 / 10 — 20%

Prev | Next

PROTEIN H2020 Questionnaire

3. Information

* 6. Select all that might apply:

- ☐ You are an athlete
- ☐ You have been diagnosed with obesity
- ☐ You have been diagnosed with diabetes
- ☐ You have been diagnosed with Cardiovascular Disease
- ☐ You have been diagnosed with a medical condition not listed above. Please specify [TEXT]
- ☐ None of the above

* 7. Who asked you to complete this survey:

- ◯ 1. OCADO
- ◯ 2. UNIVERSITY OF SURREY (UoS)
- ◯ 3. KATHOLIEKE UNIVERSITEIT LEUVEN (KUL)
- ◯ 4. POLO EUROPEO DELLA CONOSCENZA (PE)
- ◯ 5. EUROPEAN ASSOCIATON FOR THE STUDY OF OBESITY (EASO)
- ◯ 6. DIETHNES PANEPISTIMIO ELLADOS (IHU)
- ◯ 7. AGRIFOOD CAPITAL
- ◯ 8. SPORT LISBOA E BENFICA – FUTEBOL SAD
- ◯ 9. ARTICA TELEMEDICINA
- ◯ 10. ETHNIKO KENTRO EREVNAS KAI TECHNOLOGIKIS ANAPTYXIS (CERTH)
- ◯ 11. FLUVIALE
- ◯ 12. BIOSENSE INSTITUTE
- ◯ 13. CHARITE – UNIVERSITAETSMEDIZIN BERLIN
- ◯ 14. FACULDADE DE MOTRICIDADE HUMANA (FMH-UL)
- ◯ Other (please specify)

3 / 10 30%

Prev Next

PROTEIN H2020 Questionnaire

4. General

* 8. Are you aware of other apps that offer similar features (e.g., meal adaptation) compared to the PROTEIN app?

◯ No. PROTEIN is unique

◯ A few only

◯ Several Exist

◯ N/A: I don't know

Which ones (please specify)

4 / 10 40%

Prev Next

5. Evaluation

* 9. Indicate for each statement below, your level of agreement or disagreement on the provided agree-disagree scale

	Strongly Disagree	Somewhat Disagree	Neutral	Somewhat Agree	Strongly Agree	N/A
1. PROTEIN app offers something that was missing in mobile health apps	○	○	○	○	○	○
2. I found the app easy to use	○	○	○	○	○	○
3. I found the app useful	○	○	○	○	○	○
4. I am comfortable with sharing personal information on the User Profile screens	○	○	○	○	○	○
5. You are satisfied with the level of security and privacy of the app during the login process	○	○	○	○	○	○
6. The app recommended suitable ingredients/meals according to my user profile	○	○	○	○	○	○
7. The app was effective at helping me to plan healthier meals	○	○	○	○	○	○
8. The app recommended suitable activities according to my user profile	○	○	○	○	○	○
9. The app was effective at encouraging me to take more exercise	○	○	○	○	○	○
10. I feel using the app would improve my health	○	○	○	○	○	○
11. I found the automatic shopping list useful	○	○	○	○	○	○
12. I found it easy to review and organise my shopping	○	○	○	○	○	○

* 10. What are your primary health and wellbeing goals? (select all that apply)

- [ ] Eat more healthily
- [ ] Lose weight
- [ ] Be more active
- [ ] Manage an existing health condition
- [ ] Manage specific dietary requirements
- [ ] Improve energy levels
- [ ] Sports training
- [ ] Discover new healthy meal ideas
- [ ] Other (please specify)

5 / 10 50%

Prev Next

Powered by
SurveyMonkey
See how easy it is to create a survey.

6. Experience

* 11. How would you rate the amount of user interactionthe app requires?
1: Too time-consuming
5: Minimal Interaction required (fast and easy)

★ ★ ★ ★ ★

* 12. Does the user profile cover all the important information required regarding your potential allergies, deficiencies, intolerances, or personal diet goals?

1= most important attributes NOT covered
5= Everything I needed the system to consider is there

★ ★ ★ ★ ★

* 13. Do you feel the app offers sufficient variety on your meal plan?

0= No Variety, many similar meals
5= Large Variety

★ ★ ★ ★ ★

* 14. Would you regularly follow the recommended meal and activity plans?

1: Not at all likely
5: Very likely

★ ★ ★ ★ ★

6 / 10 60%

Prev Next

PROTEIN H2020 Questionnaire

7. Evaluation Part 2

* 15. What would help you to follow the recommended plan? (Select one or more)

- [ ] 1. Clearer meal suggestions
- [ ] 2. Clearer activity suggestions
- [ ] 3. Multiple options for each meal and activity recommendation
- [ ] 4. Meal suggestions that are better suited to my diet preferences
- [ ] 5. Method for how to prepare meal recipes
- [ ] 6. Options to customise the plan to my daily routine
- [ ] 7. Better goal setting and achievement tracking
- [ ] 8. More reminders
- [ ] 9. Fewer meals or activities per day
- [ ] Other (please specify)
- [ ] None of the above

* 16. How do you find recording events (meals and activity) through the app?
1: Very difficult
5: Very easy

☆ ☆ ☆ ☆ ☆

* 17. How could the app make it easier to record events (meals and activity) in the app? (Select one or more)

- [ ] 1. Improvements to the Add Meal user experience
- [ ] 2. Improvements to the Add Activity user experience
- [ ] 3. Better meal and activity recommendations
- [ ] 4. Make it easier to confirm meals eaten and activity completed on my plan
- [ ] 5. Make it easier to record meals eaten and activity completed NOT on the recommended Plan
- [ ] 6. Push notifications that remind me to record or confirm events
- [ ] 7. Better integration with activity trackers
- [ ] 8. Allow me to track activity in PROTEIN using a 3rd party app
- [ ] 9. Daily motivational features
- [ ] Other (please specify)
- [ ] None of the above

* 18. Do you feel the app is sufficiently personalised to your individual requirements and routines?
1: Not at all
5: Yes, it’s spot on

★ ★ ★ ★ ★

* 19. How could the app personalisation be improved? (Select one or more)

- [ ] 1. Option to customise the number of meal suggestions per day
- [ ] 2. Ability to select my activity preferences
- [ ] 3. Weekly activity targets vs daily activity suggestions
- [ ] 4. Make the plans easier to customise
- [ ] 5. Options to select or filter meals by cost
- [ ] 6. Filter shopping list by nutrition content
- [ ] 7. Ingredient sharing options to eliminate waste
- [ ] 8. Ability to input & track specific health goals (ie. weight loss in lbs or activity levels)
- [ ] 9. Adaptability of meal/activity plans based on daily habits
- [ ] Other (please specify)

7 / 10 70%

Prev Next

PROTEIN H2020 Questionnaire

8. Overall Evaluation

* 20. On a scale of 1-5, how motivating were the dashboard stats and notifications?

not at all motivating	a little motivating	average	quite motivating	very motivating
☆	☆	☆	☆	☆

* 21. On a scale of 1-5, how motivating did you find the point and level achievements?

not at all motivating	a little motivating	average	quite motivating	very motivating
☆	☆	☆	☆	☆

* 22. How would you rate the speed of the PROTEIN app?

Very Slow	Somewhat Slow	OK - Acceptable	Good	Very Good
☆	☆	☆	☆	☆

Please provide details of any part of the app you found particularly slow

* 23. Did you encounter any unexpected behaviour (e.g., exits) while using the app?

Very Often		Sometimes		Never - Almost Never
☆	☆	☆	☆	☆

Please provide details

* 24. How would you rate the PROTEIN app **overall**?

Very Bad	Needs Work	OK - Acceptable	Good	Very Good
☆	☆	☆	☆	☆

Briefly explain the main reason for giving this score

* 25. How likely is it that you would recommend the PROTEIN app to a friend or colleague?

Not at all likely — Extremely likely

0	1	2	3	4	5	6	7	8	9	10

26. Please provide any additional feedback on what you enjoyed MOST/ LEAST about using the application. Any other comments, improvements, bug reports or issues for this application would be most welcome

8 / 10 80%

Prev Next

9. Marketing

* 27. Would you consider paying for the PROTEIN app (if commercialised after the end of the project)?

No	Probably No	Maybe	Probably Yes	Yes
☆	☆	☆	☆	☆

* 28. Would any of the following encourage you to pay for the app - either as a one-off purchase or as subscription payment? (select one or more)

- ☐ 1. If I find the app useful after a 30-day trial period, I would pay for the app then
- ☐ 2. A large selection of healthy meal recipes
- ☐ 3. A wide selection of tailored exercise regimes
- ☐ 4. Discounts or special promotions on healthy meal purchases through using the app
- ☐ 5. Option to make shopping purchases from ANY of the main online grocery retailers
- ☐ 6. If I could get tailored plans from health experts through the app
- ☐ 7. If my doctor or health provider recommended it
- ☐ Other (please specify)

9 / 10 90%

Prev Next

PROTEIN

PERSONALISED NUTRITION FOR HEALTHY LIVING

PROTEIN H2020 Questionnaire

10. THANK YOU

Thank you for taking part in this survey, please submit your answers by clicking the 'done' button below

10 / 10 100%

Prev Done

Powered by
SurveyMonkey
See how easy it is to create a survey.
